# Genome Sequence of Lactobacillus plantarum JMCC0013, Isolated from Traditional Chinese Fermented Milk

**DOI:** 10.1128/genomeA.00407-18

**Published:** 2018-06-07

**Authors:** Shijie Wang, Lili Feng, Dong Zhang, Yuling Xue, Yiping Xun, Yuehua Ke, Hong Zhu

**Affiliations:** a Shijiazhuang Junlebao Dairy Co., Ltd., Shijiazhuang, Hebei, People’s Republic of China; b Institute of Disease Control and Prevention, Beijing, People’s Republic of China

## Abstract

Fermented food products have been consumed for thousands of years in China, so fermented Chinese foods may contain huge lactic acid bacterial resources. Here, we report the draft genome sequence of a Lactobacillus plantarum isolate, JMCC0013, collected from traditional Chinese fermented milk, which provides a precious resource for the genomic analysis of Lactobacillus strains.

## GENOME ANNOUNCEMENT

Probiotics are live microorganisms that are thought to exert a beneficial effect on the health of the consumer when administered in adequate amounts (1–3). The genus Lactobacillus represents a fundamental group among lactic acid bacteria (LAB), and most LAB have acquired “generally recognized as safe” (GRAS) status (4). Traditional fermented milk is considered a natural source for the isolation of novel probiotic organisms, which might play an important role in the prevention of infectious disease in the gastrointestinal tract of the host (5, 6). Here, we announce the draft genome sequence of Lactobacillus plantarum JMCC0013, isolated from local dairies in Dali, Yunnan, China. It has been stored in the China General Microbiological Culture Collection Center (CGMCC) as a patented strain, CGMCC no. 11727.

The genomic DNA of JMCC0013 was isolated and sequenced with an Illumina HiSeq 2000 sequencer using a paired-end protocol. The generated sequencing reads were filtered to remove low-quality reads and then assembled with Genomics Workbench version 5.5 (CLC bio) by the *de novo* assembly method. A total of 83 contigs was generated, among which the maximum length was 321,800 bp and the minimum length was 511 bp. The average length of the contigs is 19.1 kb, and the total length is 32,293,240 bp. The GC content for the contigs was 44.28%. Final approximate coverage for these contigs was about 182×.

The genome sequence was then annotated with several annotation tools. Open reading frames (ORFs) were predicted by Rapid Annotations using Subsystems Technology (RAST) (7). The rRNA was predicted by using RNAmmer (8), and tRNAs were identified with tRNAscan-SE 1.21 (9). A total of 3,257 ORFs were predicted, including 3,190 protein coding sequences, 63 tRNAs, three copies of 5S RNA, and one copy of 16S rRNA. The average length of the identified genes is 842 bp, and the total length of these genes represents 83.23% of the whole genome. In searching for the small RNA (sRNA) library, 7 sRNAs were found, the average and total lengths of which were 101 bp and 708 bp, respectively. Through comparison with the Virulence Factors of Pathogenic Bacteria (VFPB) database (10), we did not identify any protein secretion systems, which are usually thought to be key virulence factors. This implies that this strain may be safe and have the potential to be used as a probiotic bacterium. However, further detailed evaluation needs to be carried out prior to use in dairy products and administration to humans.

### Accession number(s).

The draft genome sequence and annotation have been deposited in the DDBJ/ENA/GenBank databases under the accession no. PEKI00000000. The version described in this paper is the first version, PEKI01000000. The GenBank assembly accession no. is GCA_002920935.
